# Growth and Nutrition in Pediatric Chronic Kidney Disease

**DOI:** 10.3389/fped.2018.00205

**Published:** 2018-08-14

**Authors:** Douglas M. Silverstein

**Affiliations:** Division of Reproductive, Gastrorenal, and Urology Devices, Office of Device Evaluation, Center for Devices and Radiological Health, United States Food and Drug Administration, Silver Spring, MD, United States

**Keywords:** growth, nutrition, causes, management, chronic kidney disease

## Abstract

Children with chronic kidney disease (CKD) feature significant challenges to the maintenance of adequate nutrition and linear growth. Moreover, the impaired nutritional state contributes directly to poor growth. Therefore, it is necessary to consider nutritional status in the assessment of etiology and treatment of sub-optimal linear growth. The major causes of poor linear growth including dysregulation of the growth hormone/insulin-like growth factor-I (IGF-I) axis, nutritional deficiency, metabolic acidosis, anemia, renal osteodystrophy/bone mineral disease, and inflammation. This review summarizes the causes and assessment tools of growth and nutrition while providing a summary of state of the art therapies for these co-morbidities of pediatric CKD.

## Introduction

Children with chronic kidney disease (CKD), including those with end-stage renal disease (ESRD), develop various secondary complications that significantly and adversely affect their development and quality of life. The prevalence of ESRD is about nine per one million children in the United States, with the highest incidence of new patients with ESRD appearing in early and mid-adolescence (1). Despite advances in the diagnosis and care of children with CKD, many will progress to ESRD (2). Growth failure is a major complication of CKD. Fivush et al. (3) reported that final height in about 50% of all children with ESRD will be below the 3rd centile.

## Patterns and impact of growth impairment in CKD

While growth velocity declines with more progressive CKD, impairment is observed at all levels of CKD (4, 5). As summarized by Becherucci, et al. (6), a report of the 2006 North American Pediatric Transplant Cooperative Study (NAPRTCS) study of more than 5000 children showed that about one-third achieved a final height below the third percentile, or, displayed a median height standard deviation score (HtSDS) less than −1.88. They also showed that there is a positive correlation between glomerular filtration rate (GFR) and HtSDS (4, 7, 8).

The risk for greatest growth impairment occurs if CKD begins in early childhood. Indeed, left untreated, CKD in infancy results in profound growth retardation, with a severe loss in relative height (9, 10). After infancy, growth closely correlates with GFR (11) and is most pronounced once the GFR falls below 25 ml/min per 1.73 m^2^ (12). Adolescents with CKD often display below-normal peak height velocity and final height is less than target height (13–15).

The impact of nutrition on growth is significant in any child with CKD, but is most profound in infants and young children (16). As shown by Rees et al. (16) and Mak et al. (17), poor nutrition is the most important factor contributing to growth impairment in younger children. Indeed, studies show that optimizing caloric intake in younger children with CKD and ESRD is the most effective strategy to enhance growth velocity.

Despite the significant challenges for normal growth in pediatric CKD, there have been some encouraging trends. Indeed, pubertal height gain in children with CKD/ESRD has improved (18–20). For example, a study of 384 German children receiving renal replacement therapy who were followed between 1998 and 2009 showed that children followed in the latter years demonstrated a more robust pubertal growth spurt, for which the onset was more likely to occur at the normal time period (18).

## Causes of growth impairment in CKD

Causes of growth failure include in CKD disturbances in growth hormone (GH) metabolism and insulin-like growth factor-I (IGF-I), electrolyte abnormalities, nutritional deficiency, metabolic acidosis, uremia, anemia, and inflammation (2, 16, 17, 21–23). Other hormonal changes in CKD that adversely affect growth include vitamin D deficiency, hyperparathyroidism, and hypogonadism (24–26). These factors are discussed in detail below.

### Nutritional deficiency

While vastly improved over the years, children with CKD and ESRD do experience reductions in protein, energy (also termed protein-energy wasting) and nutrient intake at all levels of CKD (27–29). Tom et al. (30) showed that in children receiving renal replacement therapy there is strong relationship between energy intake and growth. The causes of reduced intake include recurrent vomiting, anorexia and feeding problems (31). Ruley et al. (32) reported that children with CKD frequently develop gastroesophageal reflux, which contributes to reduced nutritional intake. There is evidence of reduced gastric and esophageal motility and delayed gastric emptying (33). Finally, CKD may also feature abnormal secretion and destruction of gut peptides that cause dysregulated motility, hunger, and satiety (34).

### Metabolic acidosis

As summarized by Rashid et al. (2), once stage 3 CKD develops, metabolic acidosis ensues due to a variety of renal mechanisms. Metabolic acidosis induces degradation of proteins, endogenous production of corticosteroids, and end-organ resistance to growth hormone (GH) (35). Moreover, recent studies show that metabolic acidosis (assessed by serum bicarbonate level) is associated with higher mortality in adults with CKD (36). Harambat et al. (37) studied the association between serum bicarbonate and CKD outcomes in 704 children with stage 3-5 CKD and cardiovascular disease. They found that the prevalence of metabolic acidosis was positively correlated with higher CKD stage. As seen in adults, children with the lowest quartile of serum bicarbonate level (<18 mmol/l) demonstrate the greatest risk of CKD progression and worsening secondary hyperparathyroidism.

### Secondary hyperparathyroidism and renal osteodystrophy/mineral metabolism

As summarized by Mehls et al. (38), there has long been extensive evidence of the deleterious effects of reduced renal function on bone and mineral (calcium and phosphate) metabolism in children, resulting in renal osteodystrophy (ROD). The mechanisms underlying ROD include reduced renal excretion of phosphate and impaired gastrointestinal and renal reabsorption of calcium, resulting in hyperphosphatemia and hypocalcemia, subsequently stimulating production and release of parathyroid hormone (PTH). Together, this is termed secondary hyperparathyroidism (SHPT). There are severe, harmful effects of ROD and SHPT on bone integrity and growth, most commonly displayed by fibrosis. As this process progresses, bone growth becomes impaired (39).

Besides abnormalities in calcium and phosphorous metabolism, recent evidence shows a more complicated and extensive array of biomolecular disorders causing ROD. As thoroughly summarized by Bacchetta et al. (40), early in CKD there is an increase in circulating fibroblast growth factor 23 (FGF23) levels (41). FGF23 and its co-receptor, Klotho, activate the renal FGF receptor to increase phosphate excretion. Simultaneously, FGF23 down-regulates parathyroid gland production and thereby secretion of PTH (42). As would be expected since FGF23 regulates phosphate excretion, FGF23 levels increase as renal function declines, resulting in elevated circulating levels of FGF23 in CKD, albeit with hyperphosphatemia due to reduced GFR.

Other abnormalities in mineral metabolism in CKD include reduced 1,25(OH)^2^ vitamin D (calcitriol) levels. Since 1,25 vitamin D suppresses PTH secretion, its reduced levels result in unopposed PTH secretion. Along with abnormalities in gastrointestinal function, reduced calcitriol results in impaired calcium absorption in the gastrointestinal tract (43). Unfortunately, similar to the general population, patients with CKD also suffer from 25(OH) vitamin D deficiency, further exacerbating hypocalcemia (44).

### Growth hormone/insulin-like growth factor 1 axis

The growth hormone (GH)/insulin-like growth factor (IGF)-1 axis is more complex in children with CKD. Simply, while GH has direct effects on bone growth, it mainly stimulates bone growth via IGF-1. As summarized by Ohlsson et al. (45), IGF-1 directly simulates proliferation of pre-chondrocytes, osteoblast hypertrophy, bone remodeling, and net mineralization. While serum levels of GH are normal or elevated in pediatric CKD, this phenomenon has been explained by reduced sensitivity of the bones to GH (46–48). Circulating IGF-1 levels are low, mainly due to the presence of elevated levels of circulating IGF binding proteins (49–52).

### Inflammation

Accumulating evidence strongly supports the deleterious role of inflammation in the secondary complications of CKD. A pro-inflammatory state exists early in CKD and continues throughout progression to ESRD (53, 54). Moreover, there exists a more complex condition in CKD called the malnutrition—inflammation complex. While these two phenomena interplay, it is most likely that chronic inflammation results in impaired nutrition (55–57). Pro-inflammatory cytokines such as interleukin-6 play major roles in the inflammatory process (55–60). Mechanisms underlying the malnutrition-inflammation complex include the leptin/melanocortin signaling pathway (61) and the direct effect of pro-inflammatory cytokines on muscle catabolism (62), as demonstrated in animal studies.

### Pubertal dysfunction

Children with CKD also exhibit delayed puberty, featured by hypergonadotrophic hypogonadism with elevated gonadotrophins with concomitant low-normal or reduced gonadal hormone levels (63, 64). Data shows that despite improved knowledge about the factors that cause delayed maturation in children with CKD, about 50% continue to exhibit delayed pubertal onset, with those requiring renal replacement therapy before age 13 being the most affected (18). The underlying causes include dysregulation of the hypothalamic-pituitary-gonadal axis, exhibited by impaired sensitivity to gonadotrophins and luteinizing hormone pulsatility and bioactivity (65–67).

### Reduced appetite

Rees et al. (68) raised the question of impaired appetite in CKD. As mentioned above, children with CKD do exhibit enhanced gastrointestinal disturbance, but the extent of this on appetite is hard to discern as there is great variability among the population. They suggest that the potential causes of reduced appetite such as ketosis, abnormal acid—base balance and anemia are all, to some extent, correctable; if so, appetite suppression may be treatable. Studies by Armstrong et al. (69) show that there is reduced taste sensation in CKD while other factors adversely affect appetite, including medication usage and excessive fluid intake in younger children with impaired renal concentration, and inflammation. Similarly, Mak et al. suggest an important role for inflammation on appetite in CKD (70).

### Adiponectin, resistin, and leptin

There has been extensive literature describing the essential role of adipokines (cytokines produced and secreted by adipose tissue) such as adiponectin, resistin, and leptin on nutritional status in CKD. Maggio et al. (71) measured leptin, adiponectin, resistin, glucose, and insulin levels in 31 children with CKD (mean age 12.1 ± 4.47 years) and compared the levels to those in control subjects. While glycated hemoglobin (HbA1c) levels were similar in children with CKD vs. controls, children with CKD exhibited higher levels of serum insulin, suggesting peripheral insulin resistance. Children with CKD have higher levels of serum leptin, which correlate positively with serum creatinine. Similarly, serum adiponectin levels are significantly higher in patients with CKD than controls. Finally, serum resistin levels are normal among all CKD patients, but directly correlate with C-reactive protein (a marker of inflammation). It is unclear why serum leptin and adiponectin levels are elevated, and what the impact of those levels are on appetite. However, it is possible that elevated leptin contributes to reduced appetite while higher adiponectin simulates inflammation, further contributing to the inflammation-malnutrition complex.

### Drug toxicity

Medications can have a deleterious effect on linear growth in patients with CKD. The most common drugs include corticosteroids and calcineurin inhibitors. Corticosteroids are effective drugs for some glomerular diseases, but, may adversely affect linear growth by a variety of mechanisms, including reduced pulsatile secretion of GH, impaired sensitivity of the GH receptor to IGF-1, and decreased production of IGF-1. In addition, steroid therapy may result in reduced bone density and disordered calcium-phosphorous metabolism (72).

## Assessment of body composition

There have been many studies on the nutritional status of children with CKD. Recently, Gupta et al. (73) studied nutritional intake and anthropometry in forty-five children (ages 1–18 years) with CKD. They recorded 3 days of dietary intake and collected blood to measure biochemical parameters. Sixty percent of the children had CKD stage 1, 2, or 3 while the remaining had CKD stage 4 or 5. Among the 45 children, 60% had moderate to severe malnutrition. The mean weight and height (standard deviation scores) were −2.77 ± 2.07 and −2.30 ± 1.38, respectively. They found that the prevalence of growth retardation was inversely related to glomerular filtration rate (GFR), with evidence of greater growth retardation with lower GFR. Dietary intake assessments showed that there was marked caloric deficit, with adequate protein intake but subnormal fat intake. Other vitamin and mineral deficiencies included intake of iron and calcium while there was an excess of phosphate intake. The degree of nutritional deficit was greatest in children with more advanced CKD.

As a result of an imbalanced diet and endogenous factors, children with CKD exhibit altered fat mass and lean mass (LM). This has profound consequences, as studies show an inverse and bell-shaped relationship between BMI and mortality risk (74). That said, Schaefer et al. (75) showed that when BMI is corrected for height age, its levels are only moderately raised in children with CKD. Other abnormalities in nutritional status include reduced skin-fold thickness (76) and abnormal fat distribution, with elevated truncal vs. limb fat (77). Several studies (78–80) show that there is reduced LM and high fat mass in children with CKD.

Based on the information above, it is essential to accurately assess nutritional status in children with CKD. Nutritional status in children with CKD is challenging due to the changing requirements for optimizing both physical and cognitive growth throughout childhood. Therefore, it is mandatory that the clinician adequately understand the methods to assess nutritional status.

### Body mass index

Body mass index (BMI) is a classic tool to assess body fat and nutritional status in the general pediatric population. Yet, Schaefer et al. (75) have raised concerns about the direct applicability of using certain measures of nutritional status in children with CKD. Specifically, they recommend “using height age rather than chronological age for standardization in populations suffering from growth disorders.” They cite data by Feneberg et al. (81) showing that absolute weight or raw BMI data should not be used since there are frequent and significant changes such as physical activity, dialytic therapy, and medications that have a profound effect on BMI measurement in children with CKD. This recommendation is fully supported by clinical practice guidelines (82). A study of 737 children with CKD showed that addition of waist circumference (WC) to BMI does not provide significant benefit to the assessment of the prevalence of obesity and its association with measures of metabolic, cardiovascular, and renal health in children with CKD, despite the fact there is good agreement between WC and BMI in identifying obesity (83). Finally, a cross-sectional study of 143 children with CKD and 958 healthy controls showed that compared with healthy controls, children with CKD exhibit higher BMI-age-z and LM-height-z scores. As shown by others, they found that calculating BMI relative to height-age provided greater accuracy than relative to weight-age (84).

### Skin-fold thickness

Using skin-fold thickness as a marker of fat mass may be confounded by interpreter expertise. Variables such as fluid status may result in misinterpretation of fat mass (85). Schaefer et al. (86) showed that reproducibility of skin-fold thickness measurements may be useful if the results are considered in conjunction with equations to determine whole-body percentage fat mass.

### Isotope dilution

While isotope dilution has been identified as the “gold standard” for assessing body composition, its use may be limited in CKD due to the impact of fluid on the measurement. Indeed, Wuhl et al. (87) showed that this technique may underestimate fat and LM. Moreover, although total body potassium has been used to estimate cell mass, the abnormally elevated potassium levels in muscle may result in overestimation of body cell mass (88).

### Dual energy X-ray absorptiometry

The procedure of dual energy X-ray absorptiometry (DEXA) involves the passage of two photon beams through a subject's body to create a projection of a three-dimensional structure. DEXA can provide assessment of fat and LM in children with CKD (89), using reference data. Foster et al. (90) studied the relationship between CKD and muscle mass while assessing whole body and regional LM and fat mass (FM) in children with CKD. They studied DEXA in 143 children with CKD and 958 control. They discovered that compared with controls, leg LM Z-scores were similar in CKD stages 2–3 but were lower in CKD stages 4–5 and dialysis, concluding that in more advanced stages of CKD deficits in leg LM were common.

### Bioelectrical impedance analysis

There has been excitement about the potential role of bioelectrical impedance analysis (BIA; resistance of a tissue to an electronic current) to assess nutritional status in children with CKD. Schaefer et al. (75) outlined various challenges to using BIA to assess nutritional status in CKD, including variable electrolyte content of fat-free fluids during childhood, effect of hydration status in certain diseases such as CKD, and the impact of total body water on impedance. Yet, they assessed BIA in 112 healthy children by comparing the results to a gold standard (^40^K spectrometry) and developed formulas to predict fat free mass (FFM) from BIA (86). Then, they studied the value of BIA to measure TBW, using the deuterium oxide dilution technique as a comparator, in 23 children receiving renal replacement therapy (87). They found that using the formulas they developed, BIA provided a reliable estimate of FFM in children with severe CKD. However, a study of 16 children receiving either hemodialysis or peritoneal dialysis showed that assessment of nutritional and fluid status by multifrequency bioimpedance was not as precise as dilution techniques (91).

## Management of nutritional deficiency in CKD

There is frequent reference to the term “renal diet,” but this can be misleading as the diet must be optimized and adapted for each patient. The factors that need to be considered are the patient age and gender, the current nutritional and growth parameters, stage of CKD, and the rate of progression of CKD. As comprehensively summarized by Nguyen et al. (92) and others, the major components of the diet include calories, protein, sodium, potassium, calcium, phosphorus, and iron. It is important to begin the evaluation by assessing the patient's current growth status, including height, weight, head circumference (in children up to 36 months of age), and body mass index (93) while comparing those values to available norms and adjusting for prematurity in infants less than 2 years of age.

### Caloric and energy needs

The required intake of calories (energy) should be similar to that of age-matched healthy subjects (94). The preferred modality of feeding is according to the patient age and other circumstances. Due to the vital need for adequate nutritional intake during the first 2 years of life, special attention and an aggressive nutritional plan is recommended to optimize growth and development during the early years (93). For example, infants should receive, if feasible, breast milk (which may require caloric supplements) or age-adjusted formula. Since many infants with more advanced CKD require potassium and/or phosphorous restriction, formulas low in those ingredients may be required.

If feasible, older children should begin eating age-appropriate foods but may require supplements to attain the required calories. Levitt et al. (95) recommends close monitoring of serum electrolytes if dietary supplements are prescribed.

While the goal is to generally provide energy requirements at 100% of the recommended daily allowance (RDA) for age, some children who need catch-up growth may require energy intake at 120–140% of the RDA (70).

### Modality of feeding

There are many reasons why children with CKD may not achieve adequate caloric or nutrient intake. In some patients, oral feeding may be sub-optimal, and, in such cases, feeding by nasogastric (NG) or gastrostomy tube (G-tube) may be necessary. These are both termed “tube feeding” but are not the same. While many children are initially started on NG tube feeds, there is clear evidence that G-tube feeds are generally more effective. Data from the International Pediatric Peritoneal Dialysis Network (IPPN) (96) strongly shows the superiority of G-tube vs. NG tube feeding in some children with ESRD. While growth was variable among 153 children in 18 countries receiving peritoneal dialysis, growth in children in the United States who were fed by G-tube was greater than in those fed by NG tube. Yet, there are risks and concerns associated with each modality. There is an increased risk of infection with G-tubes, especially in those receiving peritoneal dialysis (97, 98). The major disadvantages of NG feeding include the cosmetic appearance of the tube, the requirement to periodically exchange the tube, and the increased risk of developing gastroesophageal reflux (99). As shown by Ledermann et al. (97), fundoplication may be necessary in children with moderate-severe gastroesophageal reflux disease who have persistent vomiting to optimize tube-feeding.

Regardless of modality, tube feeding is designed to provide the required fluid, calories, and protein that cannot be achieved by oral feeds alone. Moreover, tube feeding may permit better medication tolerance. While some patients tolerate tube feedings well, many do experience challenges and may require gastrojejunostomy or jejunostomy tube placement (92). Finally, in patients receiving PD, G-tube placement can present complications such as blockage of the tube and leakage around the exit site with possible infection (100, 101). To reduce the occurrences of complications, insertion of the feeding tube prior to or after PD catheter placement may be necessary (98).

Lastly, it is vital to recognize that the aims of improved growth should be for height and weight gain, the former being more difficult to achieve than the latter. For example, studies show that nutritional supplementation with tube feeding more easily results in an increase in weight and BMI but not necessarily a significant increase in height, sometimes resulting in about 50% of subjects becoming overweight or obese (99, 102).

### Protein

Given the pivotal impact of protein balance on mortality (74), current KDOQI guidelines (82) recommend supplying children with stage 2–3 CKD 100–140% of the dietary reference intake (DRI) of protein for ideal body weight while children with more advanced CKD should receive 100–120% of DRI for ideal body weight. Children receiving peritoneal dialysis may require further supplementation due to excess protein loss through the peritoneal membrane. Methods to measure protein nutritional status and assess the requirements for supplementation include nitrogen balance and normalized protein catabolic rate (nPCR), which are especially important in children with CKD who are often in a hypercatabolic state (103).

### Phosphorus

Due to the prevalence of hyperphosphatemia and bone mineral disease/ROD in CKD (38), the vast majority of children with CKD require reduced intake of phosphorous-containing foods in the diet. This can be difficult to achieve since many foods contain phosphate, and, adequate phosphate intake is necessary for normal bone mineralization. Yet, the flip side of that equation is the enhanced risk for cardiovascular disease due to many factors, including hyperphosphatemia (103). As per KDOQI guidelines, dietary phosphorus intake should be 100% of DRI for age (82). However, as summarized by Nguyen et al. (92), patients with evidence of secondary hyperparathyroidism and significant hyperphosphatemia should have dietary phosphorous intake restricted to 80% of the DRI. It is essential to inform families about the content of phosphorous in foods, especially dairy and food sources rich in proteins such as meats, nuts, and beans. Finally, many patients with advanced CKD will require phosphorus binders to help manage hyperphosphatemia, which are generally effective if adherence is achieved (104). Since most phosphorous binders contain calcium, there is the risk of developing vascular calcifications in adults (105) and young adults (106) with CKD. Moreover, caution must be considered when prescribing these medications due to the risk of bone demineralization (107). There is some evidence that use of non-calcium-containing phosphate binders may reduce the development of vascular calcifications in adults with CKD (108).

### Calcium

Similar to phosphate, serum calcium plays a major role in the developing bone and is vital for proper mineralization, though the system is more complex in CKD than previously thought (109). KDOQI recommendations (82) recommend that calcium intake in children with CKD be 100–200% of the DRI for age (maximum 2500 mg of elemental calcium per day). For patients receiving calcium-containing phosphate binders, it is mandatory that the total calcium intake account for the amount of calcium in those medications (91). Patients with hypocalcemia may require supplemental calcium. As noted above, there is evidence that use of calcium-containing phosphate binders may increase the risk of vascular calcifications (105).

### Sodium

While it is common to restrict sodium in adults with CKD, the recommendations are a bit more nuanced in children since many have congenital malformations of the urinary tract as a cause of CKD, in which renal sodium wasting is common and robust. Therefore, sodium restriction in such patients must be tempered as many may require supplemental sodium, especially infants who commonly have congenital renal malformations that result in impaired sodium reabsorption (110). In contrast, children with glomerular diseases as the etiology of CKD do require sodium restriction. This commonly takes the form as a diet without added salt, resulting in restriction to 1500–2400 mg/day as per KDOQI guidelines (82).

### Potassium

Both hypokalemia and hyperkalemia predispose to a variety of secondary complications; therefore, it is essential that the diet in CKD be adapted to mitigate against disorders of potassium balance. KDOQI recommends general restriction of potassium 40–120 mg/kg/day for infants and younger children and 30–40 mg/kg/day for older children (82, 92). In addition to prescribing a diet with the appropriate amount of potassium, it is essential to provide the family with educational material that lists various foods that contain a high amount of potassium. It may be necessary in some patients to concomitantly prescribe medications to better control the serum potassium level.

### Iron

One of the more common complications of CKD in children is anemia. As summarized by Greenbaum (111), there are many causes of anemia, including a reduction in erythropoietin production and iron deficiency. The latter may be due to a variety of mechanisms including reduced intake, impaired gastrointestinal absorption, and enhanced activity of hepcidin (111, 112). Management of anemia in CKD is complex, but includes monitoring (serum iron, total iron binding capacity, transferring saturation, ferritin), with possible supplementation with iron. Supplemental oral or intravenous iron is often required but must be done with caution to avoid the perils of iron overload (113). Typical oral iron supplementation is a starting dose of 3–4 mg/kg/day of elemental iron with periodic assessment of levels (92, 114).

### Vitamins: D, B12, and folate

Vitamin deficiency in CKD may include vitamin D, vitamin B12, and folate. Vitamin D deficiency includes vitamin D (115) and 1,25(OH)^2^ vitamin D (116). Shroff et al. (116) recently published comprehensive recommendations for vitamin D (vitamins D2 and D3) therapy in children with CKD. For this publication, their extensive recommendations are challenging to summarize, but they do recommend monitoring and therapy, as needed, for vitamin D2 and D3 to maintain levels above 75 nmol/L (>30 ng/mL) in children with CKD Stages 2–5D.

Vitamin B12 and folate deficiency may result in anemia (117, 118). Folate deficiency also predisposes to hyperhomocysteinemia in children with CKD (119), predisposing patients to potential vascular complications, although the link may not be confirmed (120). However, current practice includes folic acid and vitamin B12 supplementation to pediatric dialysis patients as part of a standard water-soluble vitamin supplement.

## Growth hormone therapy for growth delay and short stature

Among other therapeutic regimens for physical growth delay, recombinant human growth hormone (rhGH) has proven effective with an acceptable safety profile for children with CKD, including patient's post-renal transplantation (8, 121, 122). A consensus committee report (123) recommended rhGH therapy in children with CKD with a HtSDS < 3rd percentile or with height velocity standard deviation score < -2 SD. They caution that rhGH therapy be started only once any nutritional and endocrinological deficits are addressed. Moreover, rhGH therapy should not be initiated in children with inadequately controlled bone and mineral disease (124).

A Cochrane database (125) systematic review and assessed the efficacy and safety of recombinant human growth hormone (rhGH) in children with CKD. They reviewed randomized controlled trials (RCTs) from the Cochrane Renal Group's Specialized Register, Cochrane Central Register of Controlled Trials from 1966 through 2011. The search criteria included children aged 0–18 years of age with CKD who received treatment with placebo rhGH. Among the 16 studies (enrolling 809 children) that met the search criteria, their research showed that treatment with rhGH (28 IU/m^2^/wk) compared with placebo or no specific therapy resulted in a significant increase in height standard deviation score (HSDS) at 1 year and a significant increase in height velocity at 6 months and 1 year. Finally, while adverse events were not necessarily prospectively collected, thereby limiting the value of that data, the authors conveyed that the frequency of reported adverse effects of growth hormone were similar in control and treated subjects.

As summarized by Mehls et al. (126), the response to growth hormone in CKD is more robust in children who start therapy at a younger age and those who receive higher doses. Their literature review showed that, in general, growth hormone improves final height by 0.5–1.7 standard deviation score (SDS) vs. the control of −0.5 SDS. Children with ESRD and those after renal transplantation also display improved growth with rhGH (127, 128).

The approach of pediatric nephrologists to GH therapy was assessed by the Midwest Pediatric Nephrology Consortium (129). They performed a cross-sectional online survey of pediatric nephrologists in the consortium and in the American Society of Pediatric Nephrology. Seventy-three pediatric nephrologists completed the survey. The main findings of their report were that about 50% of pediatric nephrologists request significant involvement by pediatric endocrinologists while the majority of those surveyed have a dedicated renal dietitian to support the program. The most common reason for withholding GH therapy to children with short stature and CKD was family refusal. There was great marked variability in the requested studies (e.g., bone age (95%), thyroid function (58%), insulin-like growth factor-1 (40%), hip/knee X-ray (36%), and ophthalmologic evaluation (7%) performed in preparation for GH therapy. This variability in practice may contribute to the lack of uniform response to GH therapy. Mehls et al. (130) analyzed data from 208 prepubertal children on conservative or dialysis treatment in a large pharmaco-epidemiological survey, the KIGS (Pfizer International Growth Database), including height velocity during the first year of GH treatment. They found that the best predictors of GH response were age at start, weight SD score, underlying renal disorder, baseline kidney function, and dose of GH. These parameters accounted for 37% of the variability of GH response.

## Summary and conclusion

There are various causes of malnutrition and inadequate linear growth in children with CKD. The most important first step to ameliorate these co-morbidities is to recognize and address their etiologies of these co-morbidities, using currently available methods and tools. It is essential to also understand the interplay between nutrition and growth. The next step is to devise an individualized treatment that requires continuous re-assessment. While advances have been made with identifying risk factors and providing improved therapy, enhanced recognition of these co-morbid conditions is essential for improved outcomes.

## Author contributions

The author confirms being the sole contributor of this work and approved it for publication.

### Conflict of interest statement

The author declares that the research was conducted in the absence of any commercial or financial relationships that could be construed as a potential conflict of interest.
